# Treatment of bipolar disorder: a complex treatment for a multi-faceted disorder

**DOI:** 10.1186/1744-859X-6-27

**Published:** 2007-10-09

**Authors:** Konstantinos N Fountoulakis, Eduard Vieta, Melina Siamouli, Marc Valenti, Stamatia Magiria, Timucin Oral, David Fresno, Panteleimon Giannakopoulos, George S Kaprinis

**Affiliations:** 1 Third Department of Psychiatry, Aristotle University of Thessaloniki, Greece; 2 Bipolar Disorders Program, Hospital Clinic, University of Barcelona, IDIBAPS, Barcelona, Spain; 3 Fifth Inpatient Department of Psychiatry and Outpatient Unit of Mood Disorders, Bakirköy State Teaching and Research Hospital for Neuropsychiatry, Istanbul, Turkey; 4 Department of Psychiatry, University of Geneva, Switzerland

## Abstract

**Background:**

Manic-depression or bipolar disorder (BD) is a multi-faceted illness with an inevitably complex treatment.

**Methods:**

This article summarizes the current status of our knowledge and practice of its treatment.

**Results:**

It is widely accepted that lithium is moderately useful during all phases of bipolar illness and it might possess a specific effectiveness on suicidal prevention. Both first and second generation antipsychotics are widely used and the FDA has approved olanzapine, risperidone, quetiapine, ziprasidone and aripiprazole for the treatment of acute mania. These could also be useful in the treatment of bipolar depression, but only limited data exists so far to support the use of quetiapine monotherapy or the olanzapine-fluoxetine combination. Some, but not all, anticonvulsants possess a broad spectrum of effectiveness, including mixed dysphoric and rapid-cycling forms. Lamotrigine may be effective in the treatment of depression but not mania. Antidepressant use is controversial. Guidelines suggest their cautious use in combination with an antimanic agent, because they are supposed to induce switching to mania or hypomania, mixed episodes and rapid cycling.

**Conclusion:**

The first-line psychosocial intervention in BD is psychoeducation, followed by cognitive-behavioral therapy. Other treatment options include Electroconvulsive therapy and transcranial magnetic stimulation. There is a gap between the evidence base, which comes mostly from monotherapy trials, and clinical practice, where complex treatment regimens are the rule.

## Background

The term 'bipolar disorder' (BD) is the contemporary label used for what is widely known as manic depressive illness, and was described for the first time by Hippocrates and Areteus. In modern times, Falret defined it as an illness in 1851. Today, two types are officially recognized, bipolar disorder type I and type II (BD-I and BD-II), and combined they account for a 3.7% prevalence rate or higher [1,2]. Both types constitute disabling conditions. Treatment aims to the resolution of symptoms, the restoration of psychosocial functioning and the prevention of relapses.

When collecting scientific data on the treatment of BD, diagnosis seems to be a problem as it is often retrospective and carries the risk of bias and memory distortions; hence it is of questionable reliability and validity.

Another problem is that while a specific treatment may be effective for the management of a specific cluster of symptoms, it may not be effective for the management of other clusters. Thus, treatment has to be regarded separately for each type of episode (manic, hypomanic, bipolar depression) and phase of the disease (acute, long-term and maintenance).

Double-blind, placebo-controlled studies are the main source of scientific proof of efficacy for available treatments. These should ideally be two-arm studies, including both the acute and the long-term (prophylactic or maintenance) phase, extending to a period of up to 6 or 12 months, depending on the investigated subtype. Nevertheless, there are no veracious data concerning all facets of affective illness.

The comparator agent is also an open issue, as it is still unclear whether this should be lithium, an antidepressant, an antipsychotic or something else, or whether the selection of the comparator agent should be based on the acute or the most recent phase. Likewise, it is still under consideration whether the ideal concept is that of a five-arm study, including a placebo and a drug-under-investigation group along with three comparator groups (lithium, antidepressant, antipsychotic). Such a concept of course is of very high financial cost, thus not yet used. The inclusion of a placebo group is of major importance [3], as its lack weakens the evidence; such a design cannot provide sufficiently accurate data because the underlying placebo response rate may be substantial and varies across, as well as within, studies. Furthermore, in the maintenance phase, the difference between placebo and an active comparator needs a follow-up period of at least 6 months, to be seen.

Another factor which may perplex the design of a clinical trial and the interpretation of its results is the fact that the patients' clinical condition and the natural history of the disease may be influenced by drug discontinuation, especially lithium discontinuation. This, especially when abrupt, is reported to elicit mania and lead to a refractory condition [4,5], thus affecting the results of a study. Age could be an additional confounding factor, as it may be responsible for an increased resistance to monotherapy [6].

Generalization of results is also a major problem. Treatments that are effective for unipolar depression are generally considered to be effective for bipolar depression as well, but not vice-versa [7]. Likewise, treatments that are effective for mania seem to be effective for hypomania as well, but not vice versa. However there is no sufficient data to support or reject these assumptions. As far as rapid cycling is concerned, data regarding the treatment of bipolar disorder in general do not necessarily apply to rapid cycling.

In this context, the development of treatment guidelines seems to be a rather important issue, in order to standardize treatment choices and apply research data to everyday clinical practice, by integrating information from different sources into easily applicable and accessible algorithms. The development of algorithms is mainly based on double-blind placebo-controlled trials, open studies and retrospective data analyses (experimental data). Expert opinion and clinical consensus is also taken under consideration, whereas consumer opinion may play an important role as well. Unlike earlier stages, which are simpler and more solidly evidence-based, as algorithms proceed to later stages, experimental data become ever more insufficient, resulting to a gradual take-over of expert opinion or clinical consensus.

Algorithms and guidelines facilitate clinical decision-making, reduce clinically inappropriate or cost-inefficient clinical practice decisions, and provide similar treatment across different settings but also a metric to assess patient response and a framework to evaluate the cost of treatment. Therefore they seem to be beneficial both for patients and the health system in general. Nevertheless, there are several potential problems associated with algorithms [8], e.g., disproportionate increase in cost-benefit ratio, biased consensus panel opinion, insufficient evidence for the development of an algorithm, poorer standard of care and inappropriate use due to a rigid, difficult to follow algorithm, sues for malpractice on the ground of deviation from an algorithm, etc.

The aim of this article is to summarize the contemporary knowledge and current practice concerning the treatment of bipolar disorder, by performing a selective review of the literature.

### Existing treatment guidelines for bipolar disorder

To date, several papers about treatment guidelines for bipolar disorder have been published [8-40]. There are also a number of guideline documents developed by national bodies that have been published. The CANMAT [37] and the NICE [34] guidelines are the most recent, but even they fail to incorporate all recent findings and approvals [41].

The gradual acceptance of the use of atypical antipsychotics such as monotherapy and of antidepressants for a limited period of time, and in combination with antimanic agents, seems to be the trend [42]. A summary of guidelines is shown in Table 1.

**Table 1:** Guidelines for the treatment of bipolar disorder

	Acute mania	Acute bipolar depression	Maintenance
TMAP, 2002	First step: Li, Vp, Olz Second step: Various combinations of two first choice agents	First step: Li, Vp, Olz, Li/Vp/Olz + SSRI/La Second step: Various combinations of two or more first choice agents, ECT	First step: Li, Vp, Olz, monotherapy or +AD (intermittent use) Second step: Various combinations of two or more first choice agents
WFSBP, 2003	First step: Li, Vp, Olz, Ris, Cbz Second step: Combinations of MS+aAPs, ECT	First step: AD+MS, SSRIs + Li/La/Vp/Cbz Second step: Combination of first choice agents, augmentation strategies, ECT	First step: After depression: AD+MS, SSRIs + Li/La/Vp/Cbz After mania: Li, MS, AP Second step: Combination of first choice agents
APA, 2002 and 2007	First step: Severe: Li/Vp+AP Mild-Moderate: Li, Vp, Olz Second step: Various combinations of two first choice agents, ECT 2007 update: Li for classic mania, Vp for mixed episodes, Cbz, Olz, Li/Vp+AP, ECT	First step: Li, La, Li+AD, ECT Second step: Various combinations of two first choice agents, ECT 2007 update: Li, Vp, La, MAOIs, SSRIs, Venf, TCAs, OFC, ECT	First step: Li, Vp, possibly Cbz, La, Ocbz. Continue the treatment proved efficient during the acute phase Second step: ECT, combination of first choice agents. AP should be discontinued 2007 update: Li, Vp, La, ECT
CANMAT, 2007	First step: Li, Vp, Olz, Ris, Quet, Arip, Zip, Li/Vp+Ris/Quet/Olz Second step: Cbz, Ocbz, ECT, Li+Vp Third step: Hal, Clpz, Li/Vp+Hal, Li+Cbz, Cloz	First step: Li, La, Li/Vp+SSRI, Olz+SSRI, Li/Vp+Bupr, Quet Second step: Quet+SSRI, Li/Vp+La Third step: Cbz, Olz, Vp, Li+Cbz, Li+Pramx, Li/Vp+Venf, Li+MAOI, ECT, Li/Vp/AAP+TCA, Li/Vp/Cbz+SSRI+La, adjunctive EPA/riluzole/topiramate	First step: Li, La, Vp, Olz Second step: Cbz, Li+Vp/Cbz, Li/Vp+Olz, Arip, Ris, Quet, Zip, Li+Ris/Quet, Li+La/SSRI/Bupr, OFC Third step: Adjunctive flupenthixol, gabapentin, topiramate, AD
NICE, 2006	First step: Severe: Olz, Quet, Ris. Li/Vp only in patients that previously responded to these agents. BZ if necessary Milder forms: Li/Vp Second step: Li/Vp+APP Third step: ECT	First step: SSRI+AM Second step: SSRI+Li/Vp+Quet, Mrz/Venf+AM Third step: ECT	First step: Discontinuation of Ads, keep Li/Olz/Vp Second step: Combinations of first step agents Third step: Combinations of first step agents plus La/Cbz

AAPs, atypical antipsychotics; AD, antidepressants; AM, antimanic agents; APs, antipsychotics; Arip, aripiprazole; BZ, benzodiazepines; Bupr, Buproprione; Cbz, carbamazepine; ECT, electroconvulsive therapy; EPA, eicosapentaenoic acid; La, lamotrigine; Li, lithium; MAOI, monoamine oxidase inhibitor; Mrz, mirtazapine; MS, mood stabilizers; Ocbz, oxcarbazepine; OFC, Olanzapine-fluoxetine combination; Olz, olanzapine; Quet, quetiapine; Ris, risperidone; SSRIs, Selective Serotonine Reuptake Inhibitors; TCA, Tricyclic antidepressant; Venf, venlafaxine; Vp, valproic; Zip, ziprasidone.

### Lithium

It is generally accepted and supported by the literature that lithium is moderately useful against all phases of BD. It is also believed to exert a specific action on suicide prevention [36,43-50] and its use is strongly endorsed by all published treatment guidelines [42]. It seems to be a somewhat more effective against classic mania (the response rate being around 40%) than against depression [36,39,51,52]. It has a relatively slow onset of action; clinical improvement generally occurs within 1 to 3 weeks of treatment.

A potential problem may be that after several years of successful use, a number of patients seem to develop a tolerance to lithium, while up to 15% of patients report a lithium discontinuation-induced refractoriness [53].

Resistance to lithium treatment could be predicted by the presence of mixed or dysphoric mania, rapid cycling, many prior episodes, poor interepisode functioning, an episode pattern of depression-mania-euthymia, comorbid substance abuse, and comorbid personality disorder [5,54]. By contrast, patients with an episodic course with euthymic intervals and the absence of rapid cycling may be better responders.

The recommended therapeutic Li blood levels for the treatment of acute mania range from 0.6–1.2 mEq/L, whereas maintenance levels could be lower, ranging from 0.6 to 0.9 mEq/L. Levels higher than 1.2 mEq/L are potentially toxic. When treating a patient with lithium, creatinine clearance is regarded to be the most reliable marker of kidney function to take into consideration.

Adverse events are to be expected during treatment with lithium [55], the most frequent being neurological, endocrinological (usually concerning the thyroid), cardiovascular, renal, gastrointestinal, hematological and dermatological manifestations and lithium intoxication. However, only about 30% of patients have more than minor complaints, whereas less than 20% of have no adverse effects at all.

### Anticonvulsants

While lithium seems to be more specific to euphoric mania, specific anticonvulsants (but not all) seem to have a broad spectrum of effectiveness, including mixed, dysphoric and rapid-cycling forms.

Valproic acid is FDA approved for the treatment of acute manic episodes. Its response rate in acute mania is around 50%, compared to a placebo effect of 20–30% [48,54,56-63]. Patients respond relatively rapidly (within 1–2 weeks and often a few days). Valproate appears to have a more robust antimanic effect than lithium in rapid cycling and mixed episodes [63,64]. Concerning bipolar depression, there is only one controlled study supporting the effectiveness of valproate [57], whereas uncontrolled data suggest that it may be less effective than against mania (response rate close to 30%) [57,65]. Although valproate seems to have significant prophylactic antimanic properties, its prophylactic antidepressant ones are low-to-moderate [65-67]. Therapeutic serum levels range between 50 and 150 mg/mL. Gastrointestinal symptoms, sedation, tremor, weight gain, hair loss, ataxia, dysarthria and persistent elevation of hepatic transaminases are among its common adverse effects.

Carbamazepine is approved by the FDA only for the treatment of bipolar mania. It is widely used, especially in continental Europe. The response rate against acute mania is close to 50% (similar to that of valproic) [68-71]. However, the response rate against bipolar depression appears to be lower (roughly 30% or less) [72,73]. Carbamazepine seems to be less effective in the prophylaxis against depressive than against manic/mixed episodes [69] and less effective than lithium [74-81]. The MAP study in 1997 [81,82] and a replication in 2003 [74] are the most important among studies comparing carbamazepine and lithium. Both studies showed a superiority of lithium over carbamazepine for the treatment of classic mania. A secondary analysis of the MAP data demonstrated that patients that don't respond to lithium may have a favourable response to carbamazepine [77], although its actual long-term efficacy is under question. The recommended dosage against acute mania is 600–1800 mg daily (blood concentration 4–12 mg/mL). Hepatic enzymes (CYP 3A4) induction occurs after several weeks, resulting to a lowering of drug levels. This may require additional upward dose titration [83]. Adverse effects are dose-related and include double or blurred vision, dizziness, sedation, ataxia, and diplopia, vertigo, gastrointestinal disturbances, cognitive impairment and hematological effects [5,84,85]. The induction of the metabolism of antidepressants, antipsychotics and other anticonvulsants is yet another major problem which makes the use of carbamazepine during combination treatment problematic.

Lamotrigine, at a daily dosage of 50–200 mg may be effective in the treatment of acute bipolar depression but not mania [45,86-93]. Moreover, it may be equally effective to lithium in the prophylaxis of any mood episode [22,45]. In depression, response rates are double than those observed under placebo (close to 50%). Lamotrigine may also be effective against rapid cycling [54]. Treatment should be initiated slowly; 25 mg daily for the first 2 weeks and then 50 mg for another 2 weeks, followed by slow increases, in order to avoid a moderately high incidence of rash.

Topiramate and gabapentin can only be used as supplementary therapy for the treatment of weight gain (topiramate) and anxiety (gabapentin), as data on them is negative [94-97]. Data regarding other anticonvulsants is not reliable. It must be pointed out that unlike antipsychotics, that seem to have a possibly antidopaminergic 'class effect' limited to the treatment of acute mania, anticonvulsants have no such effect in any phase of bipolar disorder. Each agent has a very distinct pharmacologic profile, thus should be considered separately.

### Antipsychotics

First generation (typical) antipsychotics (FGAs) are considered to be the traditional first-line treatment for acute mania, especially in Europe. TGAs, mostly haloperidol, have been used for long and are generally regarded to act faster than mood stabilizers. Nevertheless, many psychiatrists share the anecdotal clinical impression that FGAs induce depression.

Unlike FGAs, second generation (atypical) antipsychotics (SGAs) do not induce depression. Moreover, several recent studies support their usefulness in all phases of bipolar illness, either as monotherapy or as an adjunct to conventional mood stabilizers. They have a lower incidence of extrapyramidal symptoms and signs, thus considered to have a more favourable adverse effects profile. Improvement is reported to be similar among different antipsychotic agents, irrespective of whether the antipsychotic was utilized as monotherapy or adjunctive therapy [98]. Olanzapine, risperidone, quetiapine, ziprasidone and aripiprazole have already been approved by the FDA for the treatment of acute mania. These drugs are also approved for the treatment of mania in most European countries. Although available data is still limited, SGAs are considered a rather promising option for treating bipolar depression.

The use of adjunct SGAs on anticonvulsants produces a response rate increase of about 20%, while, when used as monotherapy, SGAs produce a roughly 20% difference from placebo.

Risperidone effectiveness in acute mania is supported in several studies [99] with remission rates of 42% vs 13% for placebo [85,100-107]. Dose-related extrapyramidal symptoms, weight gain, sedation and hyperprolactinemia seem to be its main disadvantages [99]. There are also a number of studies that included patients with mixed states [101,102,106].

Olanzapine has the highest number of published randomized control trials (RCTs) [1,60,85,87,108-128] and a solid basis supporting its use in bipolar disorder [129], hence it is the most well-studied atypical antipsychotic. It is approved by the FDA, but not the EMEA, for the treatment of bipolar depression (only in combination with fluoxetine), and for the maintenance phase for those patients that responded well to olanzapine during an acute manic episode [118,122,130]. Regarding mixed episodes, there are some available data, however its use is not well established. The most common adverse effects reported include dry mouth, weight gain, increased appetite and somnolence [60].

Quetiapine effectiveness in both mania and depression as monotherapy is supported by RCTs [59,69,131-139]. It is currently the only SGA approved by the FDA as a monotherapy (300–600 mg/daily) for both acute mania and bipolar depression. In depression trials, 600 mg/day were found not to be more effective than 300 mg/day. Concerning mixed episodes and rapid cycling, only some uncontrolled data is available [21,135]. The most common adverse effects include somnolence and hypotension.

The use of aripiprazole and ziprasidone as monotherapy in manic or mixed episodes is supported by existing data [42,140-144]. The most common adverse events are akathisia (Aripiprazole), somnolence and extrapyramidal symptoms (Ziprasidone).

### Antidepressants

Currently, fluoxetine, as part of the fluoxetine plus olanzapine combination, is the only antidepressant medication officially approved by the FDA for the treatment of bipolar depression [87,112,126].

In spite of the fact that there are some double-blind studies supporting their effectiveness against bipolar depression [145-147], this is still an open issue. Thus, their use and usefulness in bipolar disorder is still controversial [102]. Guidelines suggest their cautious use, always in combination with an antimanic agent [139], as antidepressants may induce switching to mania or hypomania, mixed episodes and rapid cycling [148-151]. In patients receiving a mood stabilizer, the outcome of depression could be improved by the addition of an antidepressant without significantly altering the risk of switch [152]. According to earlier studies, switching to mania or hypomania was a considerable risk, especially with tricyclics [46,153]. However, this may not apply to newer agents. Switching to mania or hypomania may occur in 7–30% of patients. This depends on the antidepressant agent and dose used and the personal (prepubertal onset) and family history [154,155]. Nevertheless, it is supported by some authors that the true rate of switching is rather low, if any [154,156-158]. The general concept however, is that dual action agents (TCAs or Serotonin and Noradrenaline Reuptake Inhibitors – SNRIs) may be more potent in increasing the risk for switching to mania or hypomania [148,159] and to development of suicidal ideation [38,160,161]. An adjunctive antimanic agent (atypical antipsychotic or anticonvulsant) may protect against switching or mixed symptoms, but this is not always the case [148,162].

A warning regarding the possible induction of suicidality (ideas and behavior but not completed suicide) by antidepressants in children and adolescents and possibly in all age groups, has been recently issued by the FDA [163], however data from the STEP-BD program does not support the idea of increased suicidality in bipolar patients treated with antidepressants [164]. Thus, this issue remains controversial.

### Psychotherapy and other non-pharmacological therapies

Hard data concerning the effectiveness of psychosocial interventions in BD are emerging. Psychoeducation is what appears to be the first line of psychosocial intervention. In bipolar patients under medication, psychoeduation, family-focused psychoeducation and cognitive-behavioral therapy seem to be the most efficacious interventions for relapse prevention. Moreover, they can help both the patient and family members to learn to recognize early warning signs of oncoming episodes, thus obtain earlier treatment interventions, and to identify possible triggering factors [165].

Although there are no definite data, the efficacy of electroconvulsive therapy (ECT) in acute mania is supported by several older clinical observations and some more recent clinical trials [166-168]. Transcranial magnetic stimulation (rTMS) of the brain at 20 Hz over the right but not left frontal cortex or 1 Hz bi-frontally is reported to be effective, however data are still insufficient and no conclusions can be drawn [169-171].

## Discussion

Previously, there has been an obvious discrepancy between recommendations made by opinion leaders and researchers and decisions made by clinicians in everyday practice. This discrepancy appeared to depict the different approaches to bipolar disorder in US and Europe, and, although today it is significantly smaller, somehow it still exists.

Treatment guidelines strongly emphasize monotherapy during the first stage of treatment algorithms. However, reality proves that this first stage is practically useless or that clinicians do not seem to appreciate it. Statistics show that the vast majority of BD patients receive more than one medication, with a significant percentage receiving three or more. Only 5–10% of patients are on monotherapy, whereas half may receive at least three different agents [172,173]. Therefore, recently, combination therapy is gaining ground even in treatment guidelines [36].

A comprehensive evaluation of the data concerning the various treatment modalities against the different facets of BD is shown in Table 2. The literature suggests that proper treatment of BD patients needs continuous administration of an antimanic agent [42], but this may be one of the reasons why depression predominates in the course of bipolar disorder.

**Table 2:** Grading of data on the basis of a modified POST method

**Agent/modality**	**Acute mania**	**Acute bipolar depression**	**Maintenance treatment**
Amisulpride	+	ND	+
Aripiprazole	++++	-	+++
Benzodiazepines	+	ND	ND
Carbamazepine	++++	++	+++
Citalopram	ND	+	+
Clozapine	++	ND	++
ECT	++	+++	+
Fluoxetine	ND	++++	++
Gabapentin	-	-	++
Lamotrigine	-	++++	++++
Lithium	++++	++++	++++
Olanzapine	++++	+++	++++
Olanzapine-fluoxetine combination	ND	++++	++
Quetiapine	++++	++++	ND
Risperidone	++++	ND	ND
Topiramate	-	+	ND
Valproiate	++++	+++	+++
Ziprasidone	++++	ND	ND
Psychoeducation	ND	ND	++++
TMS	ND	ND	ND

++++, good research-based evidence, supported randomized placebo-controlled and comparison trials; +++, fair research-based evidence, supported by randomized controlled trials but there are some drawbacks (small sample size or no placebo control); ++, some evidence on the basis of at least one small scale RCT; +, Recommendation based on prospective case studies, or large scale retrospective chart analyses and support by expert opinion; -, negative data; ND, no data.

Against acute mania, SGAs might act faster and better than lithium and anticonvulsants while their efficacy during the maintenance phase may be comparable. Quetiapine and the olanzapine plus fluoxetine combination have proven efficacy against both mania and bipolar depression. An SGA alone could be enough to control the disease manifestations in patients with a history of predominant manic or mixed episodes and rare and short depressive episodes [174]. Adding lamotrigine and increase it slowly up to 200 mg daily could help in controlling depressive symptoms. Antidepressants (mainly SSRIs), if needed, should be initiated at a low dosage with careful titration [34]. Other options for treatment-resistant patients include MAOIs, and ECT. Some authors suggest that after the second episode of bipolar illness, long term treatment is necessary and it has been claimed that maintenance treatment should last at least 2 years after an episode or 5 years if the patient has risk factors for relapse [34], however in clinical practice it is better to plan for lifetime treatment unless contraindications or specific issues argue against it.

## Competing interests

The author(s) declare that they have no competing interests.
